# Establishment and Maintenance of the Human Naïve CD4^+^ T-Cell Compartment

**DOI:** 10.3389/fped.2016.00119

**Published:** 2016-10-31

**Authors:** Susana L. Silva, Ana E. Sousa

**Affiliations:** ^1^Instituto de Medicina Molecular, Faculdade de Medicina, Universidade de Lisboa, Lisboa, Portugal; ^2^Centro de Imunodeficiências Primárias, Lisboa, Portugal; ^3^Clinica Universitária de Imunoalergologia, Hospital de Santa Maria, Centro Hospitalar Lisboa Norte, Lisboa, Portugal

**Keywords:** human T-cells, naïve CD4^+^ T-cells, naïve T-cell homeostasis, thymus, thymic activity, IL-7

## Abstract

The naïve CD4^+^ T-cell compartment is considered essential to guarantee immune competence throughout life. Its replenishment with naïve cells with broad diverse receptor repertoire, albeit with reduced self-reactivity, is ensured by the thymus. Nevertheless, cumulative data support a major requirement of post-thymic proliferation both for the establishment of the human peripheral naïve compartment during the accelerated somatic growth of childhood, as well as for its lifelong maintenance. Additionally, a dynamic equilibrium is operating at the cell level to fine-tune the T-cell receptor threshold to activation and survival cues, in order to counteract the continuous naïve cell loss by death or conversion into memory/effector cells. The main players in these processes are low-affinity self-peptide/MHC and cytokines, particularly IL-7. Moreover, although naïve CD4^+^ T-cells are usually seen as a homogeneous population regarding stage of maturation and cell differentiation, increasing evidence points to a variety of phenotypic and functional subsets with distinct homeostatic requirements. The paradigm of cells committed to a distinct lineage in the thymus are the naïve regulatory T-cells, but other functional subpopulations have been identified based on their time span after thymic egress, phenotypic markers, such as CD31, or cytokine production, namely IL-8. Understanding the regulation of these processes is of utmost importance to promote immune reconstitution in several clinical settings, namely transplantation, persistent infections, and aging. In this mini review, we provide an overview of the mechanisms underlying human naïve CD4^+^ T-cell homeostasis, combining clinical data, experimental studies, and modeling approaches.

## Introduction and Aim

The thymus provides a unique microenvironment to support the differentiation of hematopoietic progenitors into T-cells with a diverse repertoire of T-cell receptors (TCR), albeit with low self-reactivity (1). After thymus egression, the expression of the chemokine receptor CCR7 and L-selectin (CD62L) enables the naïve T-cells to patrol the body, through a continuous recirculation between the blood and secondary lymphoid organs (SLO) (2). The naïve compartment represents, therefore, the lifelong reservoir of T-cells able to mount specific responses to new antigens and to replenish the pool of memory-effector T-cells (3, 4). In addition to thymic output, several rounds of post-thymic proliferation in SLO are considered necessary to fill the peripheral naïve T-cell compartment of a growing child (5–9). Moreover, its maintenance throughout life requires a dynamic equilibrium between thymic output and peripheral homeostatic proliferation and survival, in order to counteract the naïve cell loss through death or conversion into memory/effector cells (3, 4). The understanding of the fine-tuning of these processes is of utmost importance to ensure immune competence and to promote immune reconstitution in many clinical settings, namely transplantation, persistent infections, and aging.

We review here the homeostasis of human naïve T-cells, focusing on the CD4^+^ population given its pivotal role in immune response orchestration. Data from clinical settings, experimental studies, and modeling approaches were summarized to address the establishment of the naïve compartment, and the relative contribution of thymus and peripheral mechanisms to ensure the maintenance of naïve CD4^+^ T-cells throughout life. We further outline the cumulative evidence pointing to age-dependent maturation processes in the periphery that may alter the cell threshold to homeostatic cues (2, 10–14). Finally, the existence of phenotypic/functional naïve CD4^+^ T-cell subpopulations with distinct homeostasis is reviewed (13, 15–18), with a particular focus on thymic-derived regulatory T-cells (naïve-Tregs) that given their high self-reactivity are considered essential to prevent autoimmunity (19, 20). This suppressive lineage, defined by FOXP3 expression, has recently been shown to rely on unique homeostatic requirements (19, 21).

## Establishment of the Naïve CD4^+^ T-Cell Compartment

The human thymus was shown to be active from very early in embryonic life, with reports of progenitor colonization of the thymic primordium by the eighth week of gestation and of mature T-cells featuring an already diverse TCR repertoire by the end of first trimester (22–24). Human T-cell development is a tightly controlled multistep process [reviewed in Ref. (1)], that is out of the scope of this review. αβ T-cell diversity depends on random TCR rearrangements and assembly of β- and α-chains, as well as on selection processes (1, 25). Life or death decisions are mainly dictated by the strength of TCR signaling upon interaction with peptide/MHC complexes, ultimately resulting in generation of CD4 or CD8 single-positive (SP) cells, based on MHC-II or MHC-I restriction, respectively (1, 25–27). Thymocytes expressing high-affinity TCR for self-peptide/MHC complexes are deleted by apoptosis, a negative selection process critical for self-tolerance, as attested by the multiorgan autoimmune disease associated with autoimmune regulator (*AIRE)* gene mutations that impair the expression of peripheral self-peptides by thymic epithelial cells (28, 29). Of note, self-peptide low-affinity interactions are later important for the peripheral homeostasis of the naïve compartment, by providing tonic signals that promote cell survival and low-level homeostatic proliferation (26).

The ultimate αβTCR diversity has been estimated to be as large as 10^15^ (26). It is worth emphasizing that the available tools to evaluate TCR diversity have significant limitations: spectratyping, which is based on length distribution of the most variable TCR region, poorly discriminates between loss of TCR specificities and biased clonal expansion (30, 31); and although next generation sequence (NGS) is a promising approach, the current algorithms still need improvements to fully account all variables inherent to the biology of TCR generation (25, 32, 33).

The thymus remains active until at least the sixth decade of life (34), as attested by *de novo* T-cell production in several clinical lymphopenic conditions, namely HIV/AIDS, hematopoietic stem cell transplantation (HSCT), and chemotherapy (35–38). Some authors claimed that thymic output peaks in first year of life and subsequently declines at annual rates of ~3% until 35–45 years of age, and ~1% thereafter, while others reported that the output of naïve T-cells only starts to decline in early adulthood (39, 40). This heterogeneity is, at least in part, related to differences in the methodological approaches used, namely histology versus measurement of thymic output by T-cell receptor excision circle (TREC) quantification (8, 39–43).

The DNA excised during β- and α-chain rearrangements results in several types of TRECs (44–47). sjTRECs, generated during the α-chain edition and containing signal joint (sj) sequence, have been broadly used to evaluate thymic activity (34). This PCR-based assay performed in circulating lymphocytes is also used in neonatal screening of major T-cell defects (48). TREC levels are influenced by peripheral events, namely cell proliferation and redistribution, or alterations in cell survival (41, 49). Therefore, total sjTRECs/microliter levels represent a better estimate of thymic output than sjTREC quantification within a given subpopulation, which is manifestly influenced by post-thymic proliferation (41, 49, 50). In line with this, the sj/βTREC ratio is considered a more accurate measurement of thymic activity, although the quantification of the TRECs generated during earliest TCRβ locus rearrangements is technically complex, precluding its generalized applicability (51). Since TRECs are not duplicated during mitosis, and are therefore diluted out with each cellular division (44, 52), the sj/βTREC ratio provides a good measurement of the proliferation occurring between the β and α gene rearrangement during T-cell development, a direct correlate of thymic output (41, 51, 53).

It has also been suggested that some thymocytes may egress the thymus before switching from CD45RO to CD45RA, and only acquire the typical CD45RA^+^ naïve phenotype in the periphery (54), which has implications for correctly estimating thymic output and rate of recent thymic emigrant (RTE) incorporation in the naïve T-cell pool. Moreover, there are no clear markers to identify RTEs, since while thymocytes express high levels of CD31 molecule at thymus egress, CD31^bright^ cells may persist in circulation (55–57), and not all RTEs express protein tyrosine kinase 7 (PTK7), the other suggested marker (16, 58).

Both thymic epithelial cell development defects, namely DiGeorge syndrome and FOXN1 deficiency, and defects of hematopoietic progenitors have severe clinical impact, which illustrates the non-redundant thymic contribution for the establishment of T-cell compartment (59, 60). Studies on primary immunodeficiency (61, 62), and on the immunological reconstitution achieved by the appropriate correction of these defects with HSCT (63), gene therapy (64–66), or thymus transplantation (67, 68), have been instrumental to better understand T-cell development. As an illustrative example of the knowledge that can be gathered from these clinical cases, we showed that the activity of thymus explants, evaluated by sj/βTREC ratio, drastically diminished 3 years post-thymic transplantation in a case of athymia due to FOXN1 deficiency (68). Nevertheless, this period was apparently sufficient to establish a sustained naïve T-cell compartment with a diverse TCR repertoire (68, 69).

## Thymic Versus Peripheral Contribution for Naïve CD4^+^ T-Cell Maintenance

The thymus also contributes to the maintenance of naïve CD4^+^ T-cell compartment, as demonstrated by the marked contraction observed in individuals submitted to complete thymectomy in early infancy due to corrective cardiac surgery [reviewed in Ref. (70)]. However, the thymus ability to adjust its output to peripheral requirements is still controversial, despite reports of thymic rebound in lymphopenic clinical settings (37, 71–75).

The dynamics of naïve T-cell compartment is also constrained by pressure to memory-effector differentiation (3). It has been suggested that naïve CD4^+^ T-cells may adjust their threshold for TCR activation with the length of time in circulation since thymus export (2, 8, 76) and with the aging of the individual (14, 77, 78).

Of note, in spite of the continuous environmental antigenic stimulation and the age-associated reduction in thymic output, the size of the human naïve T-cell pool features only a slight decline throughout adulthood (34, 55, 79, 80). Moreover, while sjTREC levels within CD4^+^ T-cells decrease 50–100 times with age (34, 75), the absolute numbers of naïve CD4^+^ T-cells decline only by a factor of 2–3 (79, 80). Therefore, thymic output *per se* is insufficient to guarantee the size of the human naïve CD4^+^ T-cell compartment, and a major contribution of post-thymic cell proliferation is required, as supported by *in silico* models (5–9, 81, 82). In contrast, the naïve T-cell pool in mice is almost entirely maintained by thymic output (5), emphasizing the significant differences in T-cell development and homeostasis between the two species (2, 5, 83).

Naïve CD4^+^ T-cells feature low levels of proliferation while maintaining their naïve phenotype, as demonstrated by studies using *in vivo* incorporation of deuterated water or Bromodeoxyuridine (BrdU) (7, 84, 85). It remains unclear whether naïve cell turnover changes with age (8, 77, 86, 87), as well as with the time span since thymic egress (2, 8, 76). The main proliferative cues appear to be low-affinity peptide/MHC interactions and cytokines, mainly IL-7, a γ-chain (γC) cytokine produced by stromal cells in SLO (56, 88–96). Experimental studies support that the low-affinity peptides, presented by MHC-II in a non-immunogenic fashion, are mainly self-antigens possibly related to those displayed in the thymus (97–99). In this scenario, naïve CD4^+^ T-cell proliferation could be viewed as a peripheral selection process, in which the repertoire of CD4^+^ T-cells is restricted by low-affinity self-peptides. Conversely, cytokines are thought to induce homeostatic proliferative responses without the bias of TCR specificity, and, thus, being vital to preserve broad diversity (100).

IL-7 signaling [reviewed in Ref. (101)] is strictly modulated by the expression of the α-chain of its receptor (IL-7Rα, CD127). IL-7 itself (102, 103), other γC-cytokines (102), and TCR stimulation (88, 103, 104) down-modulate IL-7Rα expression, which is upregulated in the absence of its cognate cytokine (102, 103). In adults, this cytokine was shown to preferentially drive the proliferation of CD31^+^ naïve CD4^+^ T-cells, while sustaining the expression of CD31 in a PI3K-dependent manner (21, 57). Conversely, the IL-7-driven upregulation of the antiapoptotic molecule Bcl-2 was shown to occur irrespectively of CD31 expression and to be independent of the PI3K pathway (21, 57, 105). Interestingly, CD31 engagement inhibits TCR-mediated signal transduction *via* the immune-receptor tyrosine-based inhibitory motifs (ITIMs) present in its cytoplasmic domain, raising the hypothesis that CD31 expression hampers TCR triggering, and thus favors the cytokine-driven homeostatic proliferation of CD31^+^ cells (56, 94, 106). On the other hand, TCR activation and cell division result in loss of CD31 expression (107). Therefore, the CD31^−^ naïve subset has been proposed to result and be maintained by TCR triggering with low-affinity antigens (13, 94). Accordingly, CD31^−^ naïve CD4^+^ T-cells express higher levels of the antiapoptotic BFL1/A1 than their CD31^+^ counterparts, a marker specifically induced by TCR signaling (94, 108, 109). As a result, the CD31^−^ subset proliferation is thought to cause TCR repertoire contraction, in contrast to the expected maintenance of diversity upon IL-7 driven proliferation of the CD31^+^ naïve CD4^+^ T-cell subset (94, 110).

Accordingly, the therapeutic use of human recombinant IL-7 was shown to induce preferential expansion of naïve CD4^+^ T-cells with a diverse TCR repertoire in several lymphopenia settings, namely HIV/AIDS (111, 112) and oncology (113, 114). The sjTREC content within CD31^+^ naïve CD4^+^ T-cells was reported to decrease following IL-7 administration in humans, supporting a significant degree of proliferation (114).

The maintenance of the naïve T-cell pool also depends on survival signals. During recirculation through SLO, naïve T-cells encounter IL-7, self-peptide/MHC complexes, and CCR7 ligands, all of which cooperate to produce homeostatic survival signals, namely upregulation of Bcl-2 (89, 115, 116). The relevance of cell survival pathways is further supported by the progressive loss of naïve CD4^+^ T-cells in association with defective Bcl-2 expression in patients with MST1 deficiency (117). An increase in the peripheral survival of naïve CD4^+^ T-cells in the elderly has been predicted by *in silico* models, in agreement with experimental data in mice (118).

## Naïve CD4^+^ T-Cell Heterogeneity

Naïve CD4^+^ T-cells are usually seen as a homogeneous population regarding stage of maturation and cell differentiation, although their phenotypic and functional variety is increasingly recognized (13, 15, 16, 18, 19). Next, we overview the main factors contributing to this heterogeneity, as well as the principal subpopulations identified. The paradigm of a naïve CD4^+^ T-cell population committed to a distinct lineage in the thymus is the naïve-Treg subset (19, 119), reviewed in the next section.

At thymus egress, naïve CD4^+^ T-cells feature unique phenotypic and functional properties (2, 13, 16, 76, 120). Moreover, in agreement with studies using manipulated murine models, the cell-intrinsic properties in terms of turnover, survival, and threshold for TCR activation is also modulated by prolonged time span in circulation (2, 8, 10–12, 121). It is possible that naïve CD4^+^ T-cells acquire properties before and/or after leaving the thymus that will impact on their differentiation into distinct memory-effector subsets (122).

Work from our lab and others identified two subsets within the naïve CD4^+^ T-cell compartment with distinct proliferative histories defined by the expression of CD31, which are maintained throughout life by different homeostatic mechanisms (13, 56, 57, 94). As discussed above, the proliferation/survival of the CD31^+^ subset, which includes the RTEs, is mainly driven by IL-7, whereas CD31^−^ cells proliferate in response to TCR stimulation by low-affinity self-peptide/MHC (13, 56, 57, 94). The CD31^+^ subset features a higher sjTREC content and telomere length than the CD31^−^, which has experienced more rounds of post-thymic proliferation (13, 56, 57, 94). The proportion of CD31^+^ cells within the naïve CD4^+^ compartment of human cord blood is up to 90–95% (56). Both absolute numbers and frequency of the CD31^+^ subset in peripheral blood decrease with aging, in parallel with the decline in their sjTREC content (13, 55, 56, 94, 123). In contrast, the counts of the CD31^−^ subset remain relatively constant throughout adult life despite thymic involution, leading to a progressive increase in the relative proportion of CD31^−^ within total naïve CD4^+^ T-cells with aging (13, 55, 94, 123).

In fact, age is the obvious determinant of naïve T-cell biology, impacting both on thymic activity and on SLO microenvironment where peripheral homeostatic mechanisms operate (43, 124). The functional properties of naïve CD4^+^ T-cells change along infancy, with a clear trend to differentiation into a Th2 profile of cytokine production in early life (125, 126). Additionally, thymic-generated functional populations that are hardly found beyond the first decade of life have been described with a yet unclear role in immunity, as illustrated by the IL-8-producing subset (15, 18).

Finally, the human naïve CD4^+^ T-cell compartment has been recently shown to include a small population of memory cells (<1%), as attested by rapid interferon-γ production upon TCR stimulation, which could be identified by the expression of Fas (CD95) and β-chain of the IL-2 receptor (CD122), in the absence of CD45RO (17, 18). These so-called memory-stem cells are generated during primary immune responses and are considered a reservoir for the memory pool, in line with their unique ability to self-renew through still unclear mechanisms (17).

## The Uniqueness of the Naïve Regulatory CD4^+^ T-Cell Compartment

**Figure 1:**
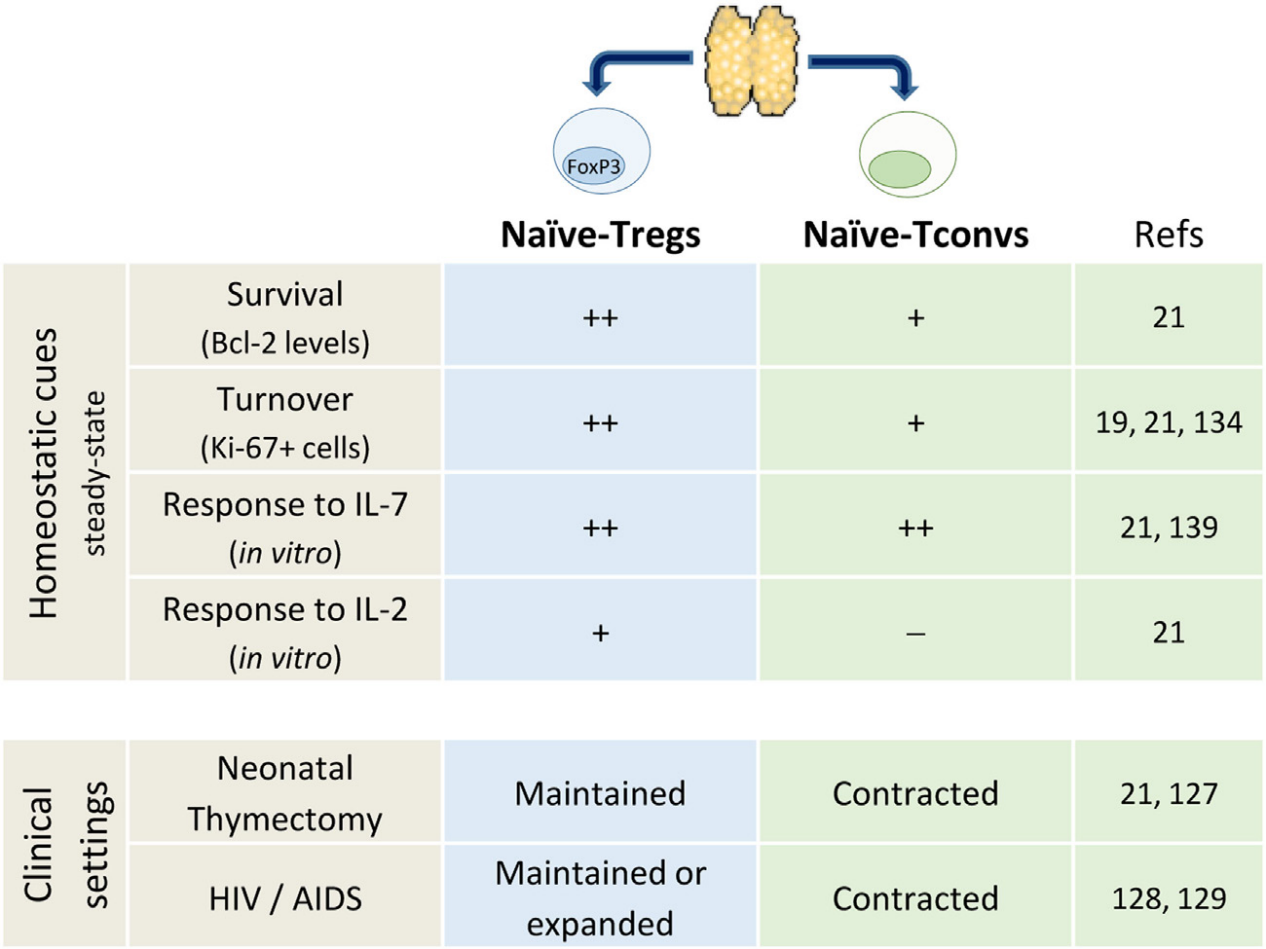
**Comparison of human naïve regulatory and conventional CD4^+^ T-cells**.

The subpopulation defined by FOXP3 expression is a hallmark of naïve CD4^+^ T-cell heterogeneity (19, 119). It is noteworthy that thymus removal early in infancy during corrective cardiac surgery was not associated with significant contraction of Foxp3^+^ naïve-Treg compartment (21, 127). Moreover, other clinical settings known to impact on thymic output and leading to conventional naïve CD4^+^ T-cell loss, namely HIV-1 infection, have been shown to feature preserved naïve-Tregs (128, 129). Altogether, as illustrated in Figure 1, these data support no thymic dependency and the existence of robust homeostatic peripheral mechanisms that ensure the maintenance of the naïve-Treg compartment, even in extreme clinical settings.

The preservation of naïve-Tregs in thymectomized and HIV-infected individuals was shown to be associated with increased turnover, as assessed by the cell cycling marker Ki-67 (21, 128). Moreover, in healthy subjects naïve-Tregs feature much higher rates of turnover than conventional naïve CD4^+^ T-cells, questioning the extent of niche sharing and competition for resources between these two naïve subsets (19, 21). Of note, matched blood and tonsil samples obtained from children submitted to routine tonsillectomy revealed higher frequency of cycling naïve-Tregs in the tonsils, with a larger fold-increase than in the conventional naïve CD4^+^ T-cell compartment (21).

T-cell receptor-affinity is thought to determine thymic Treg commitment, contributing to generate a repertoire skewed toward self-recognition (130–133). However, it has been shown that the high-affinity for self-peptides promotes their rapid differentiation into memory-Tregs upon TCR stimulation (19, 134–136), pointing to cytokines as important drivers of naïve-Treg homeostatic proliferation (21, 131).

Naïve-Tregs are known to express the α-chain of the IL-2 receptor (CD25), although at lower levels than memory-Tregs, and to similarly express reduced levels of IL-7Rα (19). Several studies have addressed the impact of IL-7 on other Treg subsets, both in human and mouse, with heterogeneous results (137–141). Our *in vitro* studies revealed that IL-7 induced PI3K-dependent proliferation, as well as Bcl-2 upregulation within naïve-Tregs, while preserving their naïve phenotype and suppressive capacity (21). Notably, their proliferation was significantly higher in response to IL-7 than IL-2 (21). Accordingly, an *in vivo* expansion of the naïve-Treg compartment was observed in patients submitted to IL-7 and to IL-2 therapy (112, 142–145).

Altogether, these data support thymic-independent maintenance of the naïve-Treg compartment (Figure 1), stressing the relevance of future research on the mechanisms counteracting the expected telomere loss and cell senescence.

## Concluding Remarks

**Figure 2:**
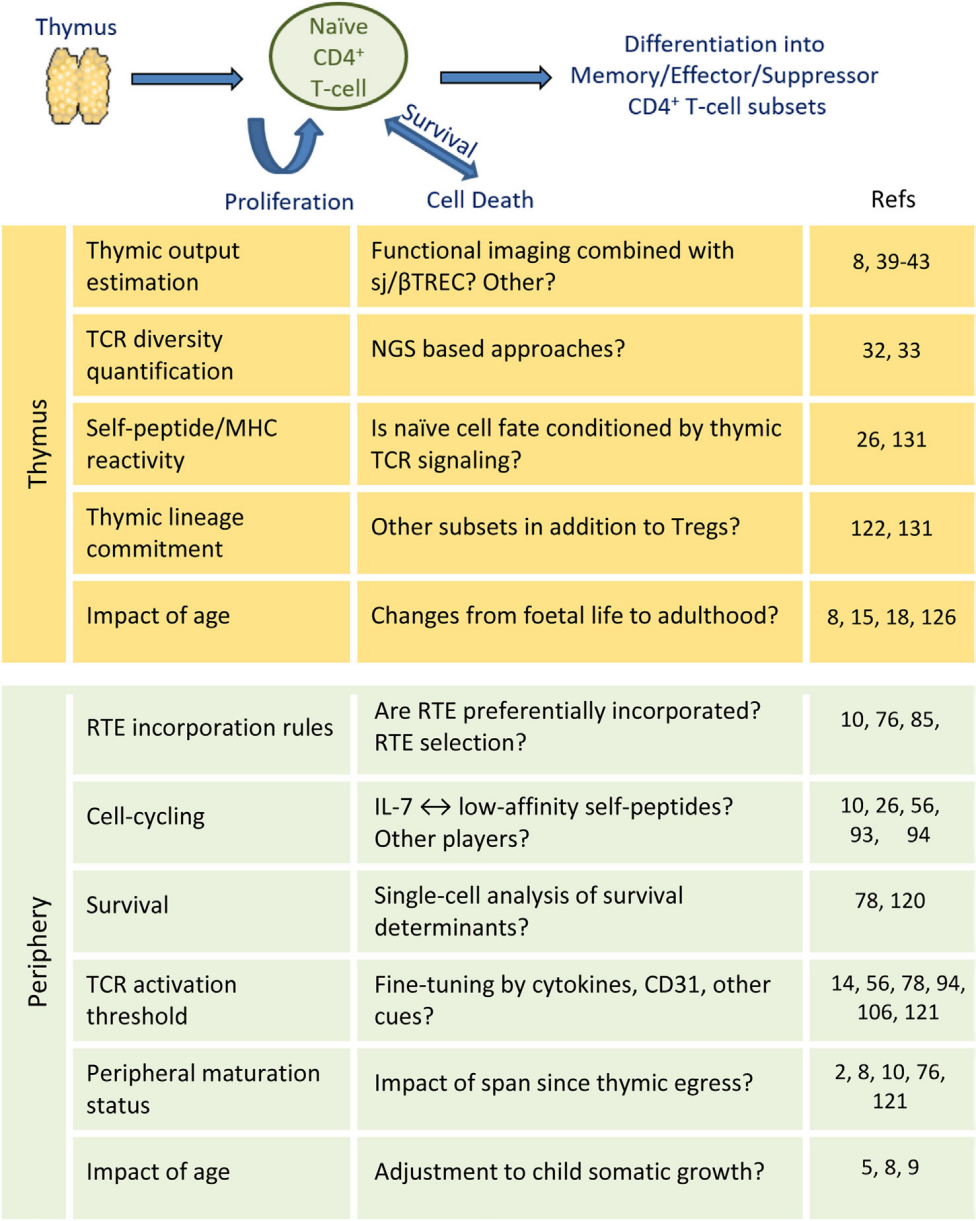
**Naïve CD4^+^ T-cell steady-state homeostasis: main open questions**.

The accelerated somatic growth and overexposure to new antigens in infancy is expected to significantly impact on size and diversity of the naïve CD4^+^ T-cell compartment, although longitudinal data are limited (146). Pediatric studies are currently facilitated by the decrease in sample size allowed by recent NGS and flow-cytometry advances (147, 148). These studies will ultimately provide a comprehensive understanding of the establishment and maturation of the naïve CD4^+^ T-cell compartment of unique value for vaccination, persistent infections, and immune reconstitution clinical settings. Figure 2 outlines the main open questions discussed in this brief review, as well as the perspectives opened by recent methodological developments. Understanding the mechanisms underlying human naïve CD4^+^ T-cell homeostasis is ultimately critical to ensure immune competence throughout life.

## Author Contributions

SS and AS prepared and wrote the manuscript.

## Conflict of Interest Statement

The authors declare that the research was conducted in the absence of any commercial or financial relationships that could be construed as a potential conflict of interest.
